# Supplementary material for: Current Technologies for Managing Type 1 Diabetes Mellitus and Their Impact on Quality of Life—A Narrative Review
Source: Life (Basel). 2023 Jul 30;13(8):1663. doi: 10.3390/life13081663 (PMC10456000; doi:10.3390/life13081663)
Supplement: Supplementary file 1 [file life-13-01663-s001.zip › life-2375819-supplementary.pdf]

Table S1. Various Insulin PPs, adapted from [86,87].

Insulin PP type	Manufacturer	Properties
Simple insulin PPs devices		
V-GO	Zealand Pharma, Zealand, Denmark	It uses relatively simple insulin dosing regimens and delivers only the basal or bolus insulin to simplify insulin therapy. It must be replaced after 24 h.
Simplicity PaQ	CeQur, Luzern, Switzerland	It holds up to 200 and 330 bolus insulin units administered in 2-unit increments and could be maintained for 3 days.
Full-Featured Electromechanical Patch Pumps		
Omnipod	Insulet Corporation Acton, MA, USA	It contains an Omnipod insulin management system, which consists of the pod and the personal diabetes manager (PDM), a controller that is wirelessly connected to the PP by Bluetooth.
Accu-Chek Solo micropump	Roche Diabetes Care; Mannheim, Germany	The PP comprises a 90-day reusable pump, a disposable 200-unit insulin reservoir, a disposable pump holder including the cannula, and a remote control.
A6 TouchCare System PP	Medtrum Technologies, Shanghai, China	It has a reusable pump base, disposable insulin reservoir, remote control, and cannula.
Panda	SFC Fluidics Fayetteville, USA.	It was supported by the 2017 "Open-Protocol Automated Insulin Delivery Systems Initiative" of the Juvenile Diabetes Research Foundation (JDRF), which aimed to establish an "open-protocol" AID ecosystem.
Sigi PP	AMF Medical; Ecublens, Switzerland	It works with readily available prefilled insulin cartridges and is controlled directly from a personal smartphone. Every user receives two long-lasting rechargeable PPs to be used interchangeably, avoiding batteries and reducing waste.
Equil PP	MicroTech Medical; Hangzhou Zhejiang China	It has a wireless portable diabetes assistant (PDA).
GlucoRx Equil	GlucoRx, Guildford, UK	It allows the user to have bolus directly or via PDA control.
Medisafe WIT	Terumo; Shibuya, Japan	Like most other PPs, the basal rate and bolus can be adjusted via remote control.
JewelPUMP	Debiotech; Lausanne, Switzerland	It has a separate controller to deliver bolus insulin doses and a reservoir with 450 units of insulin.
PPs suitable for AID systems		
Omnipod 5 system	Insulet Corporation Acton, MA, USA	Via the controlling algorithm, the PP can communicate directly with a Dexcom CGM system and a hand-held device with the Omnipod 5 App

		implemented. With this device, the user can start and stop the automated mode, deliver boluses, change settings, and view glucose data and glucose profiles.
Panda	SFC Fluidics Fayetteville, USA	Interoperable with an open protocol that allows a wireless, secure connection to other devices, such as the CGM systems or AID algorithms, in a future developing AID System made in partnership with the French company Diabeloop (Diabeloop; Grenoble, France)

PP – patch insulin pump; CGM – continuous glucose monitor; AID – automatic insulin delivery
